# Effect of Polyphenols on Oxidative Stress and Mitochondrial Dysfunction in Neuronal Death and Brain Edema in Cerebral Ischemia

**DOI:** 10.3390/ijms12118181

**Published:** 2011-11-18

**Authors:** Kiran S. Panickar, Richard A. Anderson

**Affiliations:** Diet, Genomics, & Immunology Laboratory, Beltsville Human Nutrition Research Center, Agricultural Research Service, United States Department of Agriculture, Beltsville, MD 20705, USA; E-Mail: richard.anderson@ars.usda.gov

**Keywords:** ischemia, apoptosis, inflammation, edema, glia, mPT

## Abstract

Polyphenols are natural substances with variable phenolic structures and are elevated in vegetables, fruits, grains, bark, roots, tea, and wine. There are over 8000 polyphenolic structures identified in plants, but edible plants contain only several hundred polyphenolic structures. In addition to their well-known antioxidant effects, select polyphenols also have insulin-potentiating, anti-inflammatory, anti-carcinogenic, anti-viral, anti-ulcer, and anti-apoptotic properties. One important consequence of ischemia is neuronal death and oxidative stress plays a key role in neuronal viability. In addition, neuronal death may be initiated by the activation of mitochondria-associated cell death pathways. Another consequence of ischemia that is possibly mediated by oxidative stress and mitochondrial dysfunction is glial swelling, a component of cytotoxic brain edema. The purpose of this article is to review the current literature on the contribution of oxidative stress and mitochondrial dysfunction to neuronal death, cell swelling, and brain edema in ischemia. A review of currently known mechanisms underlying neuronal death and edema/cell swelling will be undertaken and the potential of dietary polyphenols to reduce such neural damage will be critically reviewed.

## 1. Introduction

Polyphenols are natural compounds with variable phenolic structures and are rich in vegetables, fruits, grains, bark, roots, tea, and wine [1,2]. While over 8000 polyphenolic structures have been identified in plants, edible plants contain only several hundred polyphenolic structures [3]. All polyphenols contain one or more aromatic rings with more than one hydroxyl group. They are generally classified into different groups depending on the number of phenol rings and chemical groups bound to the rings [1]. Flavonoids form the largest group of polyphenols and categories of flavonoids include flavones (e.g., apigenin, luteolin,), flavonones (e.g., hesperetin,), catechins (e.g., epicatechin, epigallocatechin-3-gallate (EGCG)), and anthocyanins (e.g., cyanidin) [4]. One non-flavonoid polyphenol that has received much attention is resveratrol, a stilbene polyphenol, present in grapes and red wine with demonstrated antioxidant properties.

Diet is a major source of polyphenol intake and diets rich in fruits and vegetables contain an abundance of various classes of polyphenols. Herbs and spices, as well as wine, are also important sources of polyphenols. For instance, flavonoids including anthocyanidins are rich in blackberries, blueberries, cherries, plums, and red wine. Flavonols including myricetin, quercetin, and fisetin are found in celery, onions, spinach, apples, apricots, cranberries, dill weed, red wine, and green tea. Flavones, including apigenin and luteolin, are found in olives, fresh parsley, oregano, thyme, and rosemary. For a more detailed source of dietary polyphenols see Han *et al*. [5] and for a general review of polyphenols in human health and disease see Pandey and Rizvi [6].

Epidemiological studies support the hypothesis that consumption of diet rich in fruits and vegetables decreases the risk of cardiovascular disease, diabetes, cancer, and improves cognition associated with neurological disorders [7–9]. In addition, polyphenols also modulate immune function by reducing inflammatory cytokines in subjects with metabolic syndrome [10]. Despite such studies, an accurate assessment of the intake of polyphenols in human subjects has inherent difficulties due to several factors including inadequate information on polyphenol content in various food items, variability in polyphenol content within a food item [11], and nature of the chemical estimation methods employed [12,13]. Nevertheless, the consumption of polyphenols has been reported in several studies conducted in different countries and generally the intake of polyphenols in humans ranges from 100 mg/day to 1 g/day [14–17]. Meta analysis of prospective cohort studies have recently shown that the incidence of stroke is significantly reduced with increased consumption of polyphenols [18,19].

## 2. Oxidative Stress and Mitochondrial Dysfunction in Neuronal Ischemic Injury

Ischemic stroke is caused by an interruption of cerebral blood flow, which can lead to vascular leakage, inflammation, tissue injury, and necrosis. Changes associated with ischemia include impairment of metabolism, energy failure, free radical production, excitotoxicity, altered calcium homeostasis, and activation of proteases [20–23]. Given that the brain requires an uninterrupted supply of blood, the longer the duration of cerebral ischemia the lower the chance of reversible injury. The area which is severely affected by the lack of cerebral blood flow is termed the “ischemic core” and cell death of both neurons and astrocytes is profound in this region. The more distant region, which may be perfused with collateral arteries but yet may be in a metabolically compromised state, is termed the “ischemic penumbra”. Ironically, even though reperfusion of blood is attained after medical intervention, the restoration of cerebral blood flow is also associated with secondary neural damage and oxidative stress is one mediator of such damage [24,25]. Cerebral ischemia also causes a severe decline in the ability of brain mitochondria to function effectively thus affecting oxidative phosphorylation, a key mechanism of producing adenosine triphosphate (ATP). In addition, dysfunctional mitochondria may contribute to increased reactive oxygen species (ROS) production and would also be unable to maintain optimal mitochondrial calcium (Ca^2+^) levels which consequently can lead to depolarization of the inner mitochondrial membrane potential. Such ischemia-associated changes can contribute to Ca^2+^-induced membrane damage as well as increases in the Ca^2+^-induced proteases, free radical mediated cell damage including membrane lipid peroxidation, and DNA damage [26]. Minimizing oxidative stress and mitochondrial damage may result in reduced cell damage and a consequent improvement in cell viability following cerebral ischemia.

Cell death following cerebral ischemia is both immediate and delayed and since neurons require an uninterrupted supply of energy, any disruption of oxygen and glucose for even a short period of time can result in immediate neuronal death especially in the core region. In the penumbral region the cell death is generally delayed and the eventual outcome may well depend on the interventions and the response of the cells to such interventions. Cell death is mediated by both necrosis and apoptosis [27]. Necrosis is characterized by cell swelling and eventual loss of cell membrane integrity followed by cell lysis. Resultant inflammation is a significant part of necrotic death. In contrast, apoptosis (also known as programmed cell death) is an energy-dependent process and is characterized by a shrinking of the cytoplasm, condensation of the nucleus and eventual fragmentation of the cell body into smaller bodies. Activation of several intracellular enzymes during the process of apoptosis as well as non-random DNA fragmentation is also part of the apoptotic process. Generally, little to no inflammatory response is observed with apoptotic cell death. While apoptotic cell death is generally delayed, cells undergoing apoptotic cell death may switch to necrotic death and thus necrosis may be observed at a later time point following ischemic injury [28]. One important characteristic feature of ischemia is selective neuronal necrosis (SNN), which is characterized predominantly by neuronal death whereas the astroglial cells are spared, at least initially.

Initiation and execution of apoptotic death following ischemia is complex and diverse groups of proteins are involved in the apoptosis pathway [29] and, as discussed below, several polyphenols appear to attenuate the levels of apoptotic proteins. A family of proteins that has been implicated in an overwhelming number of studies in mediating apoptotic death is the caspases similar to *ced-3* in *C. elegans* [30]. Caspases are a family of cysteine proteases which play an important role in apoptosis, necrosis, as well as in inflammation. Sequential activation of caspases plays a critical role in the execution of the apoptotic process. Caspase-3 exists as inactive proenzyme that undergoes proteolytic processing at conserved aspartic residues to produce active subunits. This protein also cleaves and activates other caspases including caspase 6, 7, and 9 and may also itself be a target for other caspases including caspase 8, 9, and 10. Involvement of caspases in mediating mitochondrial dysfunction has been proposed [31].

DNA fragmentation is one of the hallmarks of apoptotic cell death. It occurs in response to various stimuli including oxidative stress [32,33]. Mechanisms leading to DNA fragmentation following ischemia may not be clear but a specific DNase (CAD, caspase-activated DNase) that cleaves chromosomal DNA appears to be an important enzyme in apoptotic cell death. CAD is generally found complexed with ICAD (inhibitor of CAD) which serves to limit its DNase activity. When signals for apoptosis are initiated, caspases, in particular caspase-3, cleave ICAD to dissociate CAD from ICAD, thereby allowing CAD to cleave chromosomal DNA. Another pro-apoptotic protein that is activated in ischemic injury is cytochrome C which is released from the mitochondria upon apoptotic stimuli. Release of cytochrome C can in turn activate caspase-9 which can then activate caspase-3. Another family of mitochondrial-associated proteins that have been widely studied in ischemia is the Bcl-2 family of proteins. This family of proteins consists of both pro-apoptotic (Bad, Bax, Bim) and anti-apoptotic (Bcl-2, Bcl-xl, Bcl-w) members and it is hypothesized that they exert their effects by interacting with or controlling the inner mitochondrial membrane permeability transition (mPT) pore opening. Apoptosis-inducing factor (AIF) is another mitochondrial-associated protein that is normally located in the inter membrane space of mitochondria and upon a pro-apoptotic signal AIF is released from the mitochondria. AIF subsequently migrates to the nucleus and triggers DNA damage. In addition, whether AIF also participates in the activation of caspase-9 in the cytoplasm [34] or other caspases is not clear. The relationship between AIF and caspases and the hierarchy of activation leading to apoptosis is complex but AIF has been hypothesized to function in both a caspase-dependent [35] or caspase-independent manner [36].In general mitochondrial dysfunction appears to mediate some of the critical pathways that are pro-apoptotic and preventing such dysfunction may mediate the anti-apoptotic effects.

Increased oxidative stress is a key feature of ischemic injury [37] and mitochondria are a major source of ROS [38]. Some activators of the apoptotic signaling pathway are triggered by oxidative damage and hence antioxidants have often been characterized as having an anti-apoptotic effect. One family of enzymes that are activated by oxidative stress is the matrix metalloproteinases (MMPs) [39,40] which are zinc-dependent endopeptidases that belong to a larger family of proteases known as the metzincin superfamily. While MMPs are capable of degrading extracellular proteins, they are also known to be involved in the cleavage of cell surface receptors and the release of apoptotic ligands including the FAS ligand as well as chemokine/cytokine activation [41]. In addition, MMP-9 plays an important role in the proteolytic degradation of the blood-brain barrier (BBB) after transient focal ischemia as reported in MMP-9–null mice [42].

Several studies have either shown an increased presence of apoptotic markers following ischemic injury or have reported the inhibition of cell death when using apoptotic inhibitors. The hippocampal region appears to be one of the most vulnerable regions to such injury. Also, DNA fragmentation is detected predominantly in neurons when compared to astrocytes [43,44] although oligodendrocytes are also very susceptible to cerebral ischemic injury [45]. At 3 days following ischemia, DNA fragmentation in neurons was limited to the CA1 region of hippocampus following 5 min ischemia, while DNA fragmentation was detected in CA1, CA3, dentate gyrus and cortical neurons following 10 min of global ischemia in adult gerbils [46]. Krajewski *et al*. [47] demonstrated the release of caspase-9 from mitochondria following cerebral ischemia in dogs. In a rat model of transient global ischemia, caspase-3 mRNA and protein were induced in the hippocampus which was accompanied by increased caspase-3-like protease activity [48]. In the same study caspase-3 mRNA and protein were generally increased in degenerating CA1 pyramidal neurons in the hippocampus. Also, DNA fragmentation was detected in the majority of CA1 neurons as assessed by double-label immunohistochemical studies. Ventricular infusion of Z-DEVD-FMK, a caspase-3 inhibitor, decreased caspase-3 activity in the hippocampus and significantly reduced cell death and DNA fragmentation in the CA1 sector up to 7 days after ischemia. These studies strongly implicate caspases in delayed neuronal death in the hippocampus after cerebral ischemia. Other regions where apoptotic neuronal death is observed after ischemia include the striatum and cortex but striatal response to ischemic injury appears to be an early event. Interestingly, in one study DNA fragmentation was detected as early as 3 hr after 4-vessel occlusion in the striatum in rats but was evident in the CA1 neurons only at 3 days after the insult [49]. Similarly, when time course of oxidative damage was assessed in different regions of gerbil brain after transient cerebral ischemia, lipid peroxidation was significantly increased in the striatum as early as 2 hr after the insult [50] whereas maximal oxidative damage was observed at 48 to 96 hr after the insult in the hippocampus. In the same study cerebral cortex showed early changes in oxidative stress (OS) but no significant impairment in antioxidant levels. Whether striatum is more vulnerable to ischemic injury than the hippocampus is not clear and may depend on several factors including the animal species, the injury model, and time elapsed after the initial insult. Also, whether specific brain regions are more vulnerable to ischemia-induced oxidative stress is not clear but a temporal effect appears to be involved in OS-mediated cell damage. But as seen in the hippocampal region, administration of caspase inhibitors reduces neuronal apoptosis in both striatum and the cortex For instance, transient occlusion of the middle cerebral artery augmented apoptotic neuronal death in the striatum, which was attenuated by the caspase inhibitors z-VAD.FMK and z-DEVD.FMK [51]. Cortical infarct volume was also attenuated by z-VAD.FMK in models of cerebral ischemia *in vivo* [52,53]. Likewise, administration of antioxidants or strategies that improve the antioxidant potential also attenuate neuronal death in the hippocampus [54–56], striatum [57–60], and cortex [54,59,60] following ischemia in experimental animals.

One important feature to consider is whether the type and/or severity of cerebral ischemia influences mitochondrial dysfunction qualititatively or quantitatively. Available data indicate that both focal and global cerebral ischemic models induce mitochondrial dysfunction and may further indicate a heterogeneity in mitochondrial dysfunction following ischemia [61]. In addition, a selective impairment in mitochondrial function in different subregions following complete forebrain ischemia has been reported in rats [62]. The same study showed a decline in state 3 respiration in the dorsal-lateral striatum but not in the paramedian neocortex following ischemia-reperfusion. Further, global ischemia even for a short period of time can be deleterious. For instance, global ischemia for 2 min produced a significant reduction in ATP levels in the brains of rats [63]. Other characteristics of mitochondrial dysfunction following global ischemia of different durations or ischemia/reperfusion in rats include hyperoxidation of mitochondrial electron carriers [64], decreased complex III [65], and complex I and IV activities [61], reduced pyruvate dehydrogenase complex activity (E1 component but not E2 or E3 components) [66], and the release of cytochrome c from mitochondria [67,68]. Using two-photon microscopy, a decline in mitochondrial transmembrane potential in the somatosensory cortex during ischemia and reperfusion has been reported in a mouse model of global ischemia [69]. Models of focal ischemia also induce mitochondrial dysfunction and middle cerebral artery occlusion (MCAO) has been a model of choice in mice, rats and gerbils. In rats, focal ischemia or ischemia/reperfusion has led to a decrease in ADP-stimulated and uncoupled respiration rates, with a marked fall in the respiratory control ratio [70], a transient decrease in complex IV activity [71] or a robust decline in complex IV activity [72], release of mitochondrial cytochrome c [73], potential activation of the mitochondrial permeability transition pore [74], a reduction in mitochondrial DNA content [75], depletion of mitochondrial glutathione [76], and a reversal of ATP synthetase activity [77]. Differences in mitochondrial function in the core and penumbral region of ischemia have also been reported [78]. It should however be noted that administration of 3-nitropropionic acid (3-NP), a miochondrial toxin, to rats prior to ischemia in order to induce tolerance, has resulted in decreased cerebral damage [79]. These studies indicate the complexity of cell death mechanisms activated in cerebral ischemia and implicate oxidative stress and mitochondrial dysfunction as important contributors of neuronal injury or death.

## 3. Protective Role of Polyphenols in Neuronal Ischemic Injury

A significant interest on the protective effects of polyphenols has principally been because of their antioxidant properties [80–82]. Phenolic antioxidants have been shown to inhibit the oxidation of lipids and other molecules and protect against free radicals [83]. Oxidative stress is a key event in the pathogenesis of cerebral ischemia. Overproduction of ROS during ischemia and/or ischemia/reperfusion can damage lipids, proteins, and nucleic acids, thereby inducing apoptosis or necrosis.

Increasing evidence supports the hypothesis that plant polyphenols provide protection against neurodegenerative changes associated with cerebral ischemia [84]. Whether regional differences exist in the brain in the protective effects of polyphenols in ischemic injury is not clear. Most studies have reported the protective effects of polyphenols in the hippocampal and cerebral cortex regions in ischemia. Inanami *et al*. [85] observed a dose-dependent protection against hippocampal neuronal death in ischemia in gerbils after *ad libitum* oral administration of catechin in the drinking water for 2 weeks. Epigallocatechin-3- gallate (EGCG) also protected the hippocampal region in gerbils after transient global ischemia [86] and neuronal damage in a rat model of transient focal cerebral ischemia [87]. EGCG (50 mg/kg; intraperitoneal) was effective even when it was administered 3 hr after the ischemic insult in gerbils [88]. Hong *et al*. [89] used green tea extract in the drinking water *ad libitum* for 3 weeks before ischemia in gerbils. This treatment reduced the infarct volume, the number of apoptotic cells, and lipid peroxidation, and inhibited the ischemia-induced hyperactivity. In another focal ischemia model using middle cerebral artery occlusion (MCAO) in rats, the protective effects of resveratrol were shown with pretreatment for 21 days (20 mg/kg intraperitoneally per day). The treatment reduced the infarct volume, prevented motor impairment, and inhibited lipid peroxidation [90]. A single dose of resveratrol (20 mg/kg) given orally 1 hr before permanent middle cerebral artery ligation in mice did not protect against ischemic damage. However, when given daily for 3 days before ischemia, resveratrol significantly reduced the infarct size. In another study, effects of resveratrol on transient global cerebral ischemic injury were examined in gerbils [91]. Resveratrol (30 mg/kg given intraperitoneally per day) was injected either during or shortly after common carotid artery ligation and 24 hr later. Resveratrol significantly decreased neuronal death in the hippocampus and also inhibited glial cell activation. Nanocapsule encapsulated quercetin treatment resulted in significant protection to endogenous antioxidant enzymes against ischemia induced oxidative damage in neuronal cells of young and old rats [92]. Not many studies have reported the effects of polyphenols in the striatum except that by Shukla *et al*. [93] who saw a significant inhibition in lipid peroxidation and an increase in superoxide dismutase (SOD) activity in corpus striatum in rats pre-treated with curcumin prior to MCAO. Some studies have examined the protective effects of polyphenols in the striatum but not in ischemic injury. For instance, GTE and EGCG were effective in preventing the depletion in striatal dopamine and tyrosine hydroxylase protein levels in a mouse model of Parkinson’s disease [94]. Likewise, several additional studies have also reported a beneficial and/or protective effect of polyphenols in the striatal region of animals following various conditions including aging [95,96], oxidative stress [97,98], and in a mouse model of Alzheimer’s disease [99]. It appears from these studies that protective effects of polyphenols would potentially be observed in the striatal region if assessed in ischemic injury. As mentioned above, cerebral cortex is another region where ischemic injury has been observed. Shukla *et al*. [93] reported an antioxidant effect of curcumin in the cortex of rats subjected to MCAO. Red wine polyphenol compounds also protected against oxidative stress in rats following MCAO/reperfusion [100]. Similarly, resveratrol significantly attenuated neuronal death in and decreased the generation of ROS, lipid peroxidation and nitric oxide (NO) content in the cortex of rats subjected to transient global ischemia [101]. 2,3,5,4′-tetrahydroxystilbene- 2-O-beta-D-glucoside (TSG), an active component of the rhizome extract from *Polygonum multiflorum*, significantly reduced infarct volume in the cortex following MCAO [102]. Taken together, these studies indicate that polyphenols either exert or have the potential to exert neuroprotective effects in various regions in the brain that are vulnerable to ischemic injury. While the precise dose required to achieve a neuroprotective effect in cerebral ischemia is not clear and may vary with individual polyphenols, Sutherland *et al*. [87] have reviewed the effects of green tea catechins including the safety and efficacy of such catechins.

One mechanism underlying the neuroprotective effect of polyphenols is possibly through its effects on reducing the levels of apoptotic markers. Pomegranate polyphenols and resveratrol protect neonatal mouse brain from ischemic injury by reducing caspase-3 and calpain activation [103]. In neonatal rats, amentoflavone blocked the activation of caspase-3 and the proteolytic cleavage of its substrates following hypoxic-ischemic injury [104]. Pomegranate juice also diminished caspase-3 activation in the hippocampus and cortex of the neonatal brain against a hypoxic-ischemic insult through supplementation of the maternal diet with pomegranate juice [105]. Mangiferin and morin, two antioxidant polyphenols, are neuroprotective in both *in vitro* and *in vivo* models of ischemia possibly by reducing Ca^2+^ influx and decreasing caspase-3 [106]. A subsequent *in vitro* study by Campos-Esparza *et al*. [107] demonstrated that mangiferin and morin reduced the formation of ROS and restored the mitochondrial membrane potential following excitotoxic stress, which is a major component of ischemic injury. Further, these polyphenols also reduced the glutamate-induced activation of calpains, normalized the level of cytosolic Bax and inhibited the release of AIF from mitochondria. These actions of mangiferin and morin could well be part of their profile in an *in vivo* model of ischemic injury. EGCG, a green tea polyphenol, reduced up-regulation of MMP-9 activity and neuronal damage following transient focal cerebral ischemia in C57BL/6 mice [108]. MMP-9 downregulation by resveratrol was also observed in an *in vitro* model of neuronal ischemic injury [109]. 5,7,3′,4′,5′-pentahydroxy dihdroflavanol-3-*O*-(2″-*O*galloyl)- beta-*d*-glucopyranoside (AP1), a polyphenolic compound isolated from *Anogeissus pendula* Edgew (an arid forest tree), was effective in reducing apoptotic cells in rat brain following transient focal cerebral ischemia [110]. The effect of TSG in protecting rat brain from MCAO is by increasing the anti-apoptotic Bcl-2 proteins [102]. Curcumin, a potent polyphenol antioxidant enriched in turmeric, reduced cytochrome c release and subsequent caspase-3 activation following global cerebral ischemia in Mongolian gerbils [111]. While the aforementioned studies have demonstrated a decrease in caspase-3 levels in the presence of polyphenols, it is unclear whether polyphenols act directly on caspase-3 or whether they act on upstream caspases that are precursors to caspase-3. Alternatively, such polyphenols could also be activating inhibitor of apoptosis (IAP) which would then inhibit caspase-3 activation. In addition, effects of polyphenols may also involve protecting mitochondrial dysfunction in ischemic injury as seen *in vitro* [112,113]. Preventing the decline in mitochondrial membrane potential following ischemic injury may subsequently confer protection against apoptotic cell death. In addition, resveratrol can induce neuroprotection by increasing mitochondrial ATP synthesis efficiency in rat brain following ischemia [114]. While these studies highlight the potential neuroprotective mechanisms by which polyphenols attenuate cell death in ischemia, their antioxidant and anti-inflammatory effects may also contribute to their ability to reduce cell swelling and/or brain edema which can be deleterious to neuronal and glial functioning.

## 4. Oxidative Stress and Mitochondrial Dysfunction in Brain Edema and Cell Swelling in Ischemic Injury

Brain edema is a key component of ischemic injury [115]. It is characterized by an abnormal accumulation of fluid in the brain parenchyma resulting in a volumetric enlargement of cells or tissue. The early development of cerebral edema is an important clinical consequence following ischemia since edema can lead to increased intracranial pressure, brain herniation, irreversible brain damage, and ultimately, death. Brain edema is generally classified into cytotoxic or vasogenic edema [115,116]. Cytotoxic edema is defined as a cellular swelling with fluid accumulating within the cell. Astrocyte (glial) swelling is a major component of cytotoxic edema [117]. Vasogenic edema is characterized by a breakdown of the BBB resulting in increased fluid accumulation; originating from blood vessels that amass around cells. Entry of serum proteins into brain parenchyma, normally limited by the tight endothelial cells of the BBB, is a key feature of vasogenic edema. Cytotoxic edema is a relatively early event when compared to vasogenic edema [118,119] and secondary damage to cells surviving the primary ischemic insult may be reduced by suppressing brain edema [120]. In addition, the ability of swollen and dysfunctional astrocytes to effectively clear glutamate and K^+^ from the extracellular regions may be hampered and may further contribute to excitotoxicity [23,121]. A reduction in edema may also improve neurological outcome [122] and cerebral microcirculation [123,124]. Therefore, interventions that reduce edema and associated secondary damage may play a role in diminishing the severity of ischemic injury [125]. Both vasogenic and cytotoxic mechanisms contribute to overall ischemic brain edema [115,126] and consequently impair cerebral perfusion and oxygenation that aggravate ischemic injuries. Another form of edema that has been observed in animal models of ischemia is the interstitial edema [127–129]. Interstitial edema occurs due to the rupture of the barrier of cerebrospinal fluid (CSF) and brain. This rupture can result in the transependymal flow of CSF which then allows the CSF to penetrate the brain and occupy the extracellular space of white matter. One important difference between interstitial edema and vasogenic edema is that unlike vasogenic edema such fluid does not contain any significant amount of protein. Currently, there is a paucity of agents to attenuate brain edema effectively.

Astrocyte swelling is a major component of cytotoxic edema and can be deleterious when such swelling prevents astrocytes from carrying out critical functions such as maintenance of ionic homeostasis, prevention of excitotoxicity, scavenging free radicals, provision of nutrients and growth factors, promotion of neovascularization, and support of synaptogenesis and neurogenesis that potentially may influence the outcome of ischemic injury. While such astrocyte swelling can be deleterious, loss of key astrocytic functions may also be an important factor in neuronal injury/death [23]. In addition to astrocytes, neurons [130,131] as well as oligodendrocytes [45,132] also swell in the brain in the acute phase of ischemia but such swelling may be followed by cell shrinkage and/or cell death. The relative contribution of each of these cell types to brain edema is not clear since neurons and oligodendrocytes are more susceptible to cell death than astrocytes. Given that astrocytes outnumber neurons, swelling of astrocytes likely plays a predominant role in cytotoxic brain edema.

Breakdown of the BBB is a key feature of vasogenic edema and reperfusion may further increase the damage to the BBB [133,134]. At the interface between blood and brain, endothelial cells and associated astrocytic foot processes form “tight junctions.” The tight junction is composed of smaller subunits that are transmembrane proteins including occludin, claudin, junctional adhesion molecule (JAM), and endothelial cell–selective adhesion molecule (ESAM). Each of these transmembrane proteins is anchored into the endothelial cells by the scaffolding protein complex that includes zonula occludens-1 (ZO-1), a membrane associated guanylate kinase homologue protein, and related proteins ZO-2 and ZO-3. Around the endothelial cells is a basal lamina composed of extracellular matrix proteins. Taken together the whole unit is also referred to as the “neurovascular unit” [135]. As discussed below, disruption to the BBB can occur as a result of damage to any number of elements that are involved in the composition of the neurovascular unit.

Mechanisms underlying cytotoxic brain edema are not clear [136–138] although a number of factors have been implicated in astrocyte swelling [139]. These factors include increased free radical production, increased levels of Ca^2+^, [140], elevated K^+^ [117], acidosis, release of excitatory neurotransmitters, especially glutamate [141,142] and mitochondrial dysfunction, in particular the opening of the mitochondrial permeability transition (mPT) pore [143–146]. The mPT is characterized by an increase in permeability of the inner mitochondrial membrane to small solutes (ions and molecules, reducing equivalents) [147,148]. The mPT is associated with the depolarization of the inner membrane potential (ΔΨ_m_) and may lead to osmotic swelling of the mitochondrial matrix [149], mitochondrial dysfunction, defective oxidative phosphorylation, impaired ATP synthesis, and the generation of free radicals [150] which can subsequently lead to cell swelling. Cyclosporin A (CsA), an immunosuppressant, is a relatively specific blocker of the mPT, but it also inhibits calcineurin. Sensitivity to CsA, but not to FK506, another immunosuppressant that does not block the mPT but inhibits calcineurin, is generally indicative of the involvement of mPT [151,152]. Using such a combination of CsA and FK506 to implicate the mPT, it has been suggested that blockade of the mPT attenuates glial swelling in ammonia neurotoxicity [144], *in vitro* trauma [145,146], and ischemic injury [112,113,143].

Ion transporters or exchangers play an important role in the development of cytotoxic edema and their function can be modified by oxidative stress. One important co-transporter involved in cell volume regulation in glial cells is NKCC, a membrane protein that transports Na^+^, K^+^ and Cl^−^ ions across the cell membrane. In states of high K^+^, such as after ischemia, activation of the co-transporter leads to astrocyte swelling/brain edema, and such edema is significantly attenuated by bumetanide, an inhibitor of NKCC [139,153]. Likewise, astrocytes from NKCC-deficient mice show less K^+^-induced swelling [154]. Other ion exchangers that are involved in cell volume regulation are the Na^+^/Ca^2+^ exchanger (NCX) and Na^+^/H^+^ exchanger (NHE) (see 138 for review). Another ion channel that has been demonstrated to mediate brain edema in ischemia is the SUR-1 regulated NC_Ca-ATP_ channel [155,156]. This channel conducts all inorganic monovalent cations, but is impermeable to Ca^2+^ and Mg^2+^, although Ca^2+^ is required for the opening of this channel. Depletion of ATP triggers channel opening. Blockade of this channel using glibenclamide reduced cerebral edema *in vivo* in two models of ischemia [156]. Another membrane protein that is critically involved in brain edema is aquaporin 4 (AQP4) [157] although other aquaporins (AQP1 and AQP9) have been associated with increased edema following ischemia [158]. Aquaporins are membrane proteins that transport water bi-directionally in and out of the cells selectively while generally preventing the passage of other ions or solutes [159]. Generally found in astrocytic end feet, they are also found in endothelial cells [160]. AQP4-deficient mice show reduced brain edema after acute water intoxication and ischemic stroke [157]. It is not clear whether aquaporins work alone or if they work in co-ordination with an inward rectifying K^+^ channel, Kir4.1, to reduce swelling [161,162].

The functional integrity of the BBB is critical in maintaining normal brain volume and disruption of the BBB contributes to vasogenic edema. As with cytotoxic edema, oxidative stress is an important factor in inducing vasogenic edema. Swelling of the astrocytic end feet, which are rich in AQPs as well as other ion transporters including NHE, may create a leaky environment leading to compromised microvascular integrity. Activation of MMPs, which are increased after ischemic injury [163], disrupt the BBB. Tight junction proteins (TJPs), occludin and claudin-5, which form the endothelial barrier, are vulnerable to attack by MMPs. Basal lamina proteins of the extracellular matrix proteins, such as fibronectin, laminin, and heparan sulfate, are also degraded by MMPs. Another feature of ischemia is the migration of neutrophils from the periphery to the area of ischemic lesion in the brain [164]. While neutrophils themselves can cause edema by infiltrating into the tissue, they are also a source of free radicals, MMPs, and myeloperoxidases that can aggravate endothelial damage and thus contribute to vasogenic edema. Mediators of inflammation, including TNF-α, IL-1β, ICAM-1, augment endothelial dysfunction. In addition, oxidative and nitrosative stress can also disrupt BBB integrity through mechanisms that are not clear, considering the abundance of proteins that are involved in the structure and function of the BBB.

## 5. Role of Polyphenols in Attenuating Oxidative Stress and Mitochondrial Dysfunction in Brain Edema and Cell Swelling

Oxidative stress is a key component of ischemic injury including cell swelling and brain edema and polyphenols, due to their antioxidant properties, would be postulated to attenuate such injury. Reports on the beneficial effects of polyphenols on brain edema in ischemia are scarce. Resveratrol has been reported to reduce brain edema in rats following MCAO [165]. Lee *et al*. [88] reported a protective effect of green tea polyphenol EGCG against neuronal damage and brain edema after unilateral cerebral ischemia in gerbils. AP1, a polyphenolic compound, also reduced brain edema in rats after transient focal ischemia [110]. We recently reported the protective effects of polyphenols from green tea [112] as well as cinnamon [113] on glial swelling in cultures following ischemia-like injury [138]. Myricetin and quercetin also attenuated cell swelling following oxygen-glucose deprivation in C6 cultures [166,167]. While in cell culture studies polyphenols reduced cell swelling, it is possible that the reduction in cell swelling was not due to the antioxidant effects of polyphenols. Firstly, known antioxidants were not effective in reducing cell swelling following ischemic injury [112,113]. For instance α-tocopherol, a lipid peroxidation inhibitor, prevents glial swelling in cultures when exposed to pathological concentrations of ammonia [168] or *in vitro* traumatic brain injury (TBI) [146], but not oxygen-glucose deprivation (OGD) [113]. But several possibilities exist for α-tocopherol not having a protective effect. One possibility is that different cellular mechanisms underlie cell swelling in OGD when compared with ammonia toxicity or TBI in cultures and that lipid peroxidation is not a major contributor to cell swelling in OGD. This is somewhat surprising due to similarities between ischemic injury and TBI [169,170]. Noteworthy is the differential effect of inhibition of lipid peroxidation that protects against neuronal damage after transient but not permanent middle cerebral artery occlusion (MCAO) in rats [171]. Another possibility is the species of free radicals that dominate cell swelling in OGD are different from those seen in ammonia neurotoxicity or TBI and need to be investigated in future studies. Resveratrol, another polyphenol which also has antioxidant effects [172] also did not prevent cell swelling following ischemic injury [113]. Secondly, concentrations of quercetin that significantly attenuated free radical production in C6 glial cultures did not reduce OGD-induced cell swelling [167]. Thirdly, although cinnamon polyphenol extract (CPE) reduced cell swelling following OGD in cultures [113] CPE also increased nitric oxide. Donors of nitric oxide increase astrocyte swelling in cultures [168]. These studies indicate that the precise role of oxidative (or nitrosative) stress is not clear. Yet, OS may be a contributing factor in cell swelling or brain edema and it is possible that multiple species of free radicals have to be blocked together to see a protective effect.

It is possible that different species of free radicals further activate diverse downstream molecules, including kinases, which may possibly contribute to cell swelling/edema. In such a case, one potential mechanism by which oxidative or nitrosative stress would induce cell swelling/edema is through the activation of intracellular signaling kinases [173,174]. The premise here is that the oxidative and nitrosative stresses would activate such kinases in a very short period of time and subsequently be inactivated themselves prior to the antioxidants exerting their potential effects. However, the activation of kinases would be sufficient to initiate downstream events that lead to cell swelling. Several intracellular signaling kinases have been implicated in glial swelling or brain edema. Blockade of c-Jun N-terminal kinase (JNK), in combination with hyperbaric oxygen reduced brain edema in rats after MCAO [175] or with JNK inhibitor alone following subarachnoid hemorrhage in rats [176]. Inhibition of sphingosine kinase (SphK), with the SphK inhibitor dimethylsphingosine, reduced brain edema in hypoxic preconditioned mice subjected to MCAO [177]. A novel small molecule, KDR kinase inhibitor Compound-1 (an inhibitor of kinase insert domain receptor (KDR, a vascular endothelial growth factor receptor), was effective in reducing edema following focal cerebral ischemia in rats [178]. Intraventricular administration of SB 203580, an inhibitor of p38^MAPK^, significantly attenuated BBB extravasation and subsequent edema (vasogenic) after transient focal ischemia in rats [179]. Increased phosphorylation of the tyrosine kinase p125 focal adhesion kinase (p125(FAK)) and p38^MAPK^ has also been reported in ischemic chicken retinal swelling [180] and blockade of Src kinases reduces retinal edema in mice [181] indicating an important role of kinases in the development of edema. While it is not clear how the activation of such kinases could regulate cell volume, one potential mechanism is through their subsequent phosphorylation of ion channels/transporters and modifying their action [182,183].

The effects of polyphenols in regulating ion channels or ion co-transporters, following cerebral ischemia, are not clear, and are a subject of current investigation. An increase in intracellular calcium is a key feature of ischemic injury [184,185]. Further, an increase in [Ca^2+^]_i_ can induce cell swelling as demonstrated in lactacidosis-induced glial swelling [186,187] and in hypo-osmotic swelling in cultured astrocytes [188]. Studies on the direct effects of polyphenols on ion channels or co-transporters involved in regulating intracellular calcium and thereby affecting cell volume regulation are lacking. We have demonstrated that quercetin and myricetin both attenuate OGD-induced increase in [Ca^2+^]_i_. Also, such blockade of the rise in [Ca^2+^]_i_ by blockers of the L-type calcium channel as well as modulation of [Ca^2+^]_i_ through BAPTA, a calcium chelator, reduces cell swelling in C6 glial cultures [167]. Other studies have also shown a decrease in [Ca^2+^]_i_ following administration of polyphenols. Quercetin attenuated the H_2_O_2_-induced calcium dysregulation in PC12 cells [189]. Quercetin, catechin, and resveratrol also inhibited cardiac voltage gated sodium channel in rat cultured myocytes, but had no effect on the reverse mode NCX, the Na^+^/Ca^2+^ exchanger [190]. Apple condensed tannins inhibit the increase in intracellular free Ca^2+^ concentration in RBL-2H3 cells induced by antigen stimulation [191]. EGCG reduces the glutamate-induced [Ca^2+^]_i_ increase by attenuating ionotropic Ca^2+^ influx in PC12 cells [192]. In contrast, elevated [Ca^2+^]_i_ was observed after exposure to tannins in HL-60 cells [193]. Red wine polyphenols increase calcium in bovine aortic endothelial cells [194]. EGCG increases [Ca^2+^]_i_ in U87 cells by influx of extracellular Ca^2+^ as well as by releasing calcium from intracellular stores. Whether this difference in modulating [Ca^2+^]_i_ by polyphenols is due to the difference in cell types or the polyphenols tested or the stressor involved is not clear. It is also possible that the difference arises from assessing [Ca^2+^]_i_ in control conditions (in the absence of stress) compared to examining the effects of polyphenols in the presence of stress. Nevertheless, these studies indicate that polyphenols have the potential to modulate calcium channels that are involved in cell volume regulation, but their role in attenuating glial swelling/cytotoxic edema in ischemia needs to be further elucidated.

Mitochondrial dysfunction is an important characteristic of ischemia [62,195]. The mitochondrial permeability transition (mPT) has been implicated as one mechanism, or at least part of the mechanistic pathway, for cell swelling in cultured astrocytes following ammonia toxicity [144] or TBI [146] as well as in brain sections in ischemia [143]. Despite these studies, the role of the mPT in cell swelling is not clear. We demonstrated that the attenuation of cell swelling and the prevention of the decline in mitochondrial inner membrane potential (ΔΨ_m_) by immunosuppressants, cyclosproin A (CsA), but not FK506, are consistent with the role of the mPT mediating such events. Similar to CsA, CPE, and green tea polyphenols, also significantly prevented OGD-induced cell swelling and the decline in ΔΨ_m_ in C6 glioma indicating that one mechanism by which CPE and GTE exert their protective effects is possibly by blocking the mPT. Interestingly, quercetin significantly attenuated cell swelling in C6 glial cells following OGD but did not block the dissipation of the ΔΨ_m_ [167] indicating that other factors, besides the mPT, mediate the development of cell swelling in ischemic injury. It is also possible that preventing the induction of the mPT may be sufficient in some cases but may not be always necessary. Whether this depends on the severity of the injury is not clear and needs to be investigated.

An increase in inflammatory markers has been associated with brain edema [196] and could potentially cause damage to the BBB [197,198]. A disruption of the BBB is observed in vasogenic brain edema. A key characteristic of polyphenols is their anti-inflammatory property [199,200] and anti-inflammatory effects of polyphenols have been reported in cerebral ischemia [104,110,201]. Inflammatory molecules can damage mitochondrial function. For instance, exposure of rat astrocyte cultures to interferon −γ, in the presence or absence of LPS, can increase NO production which can subsequently damage the mitochondrial respiratory chain complex function [202]. Studies that investigated the role of polyphenols on BBB function in ischemia are scarce except that reported by Zhang *et al*. [203] which examined the effects of green tea polyphenols on BBB permeability following MCAO in rats. They report a decrease in BBB permeability in the ischemic region in the presence of green tea and a concomitant decrease in levels of caveolin-1, a protein involved in BBB functioning and permeability. Wang *et al*. [165] and Lee *et al*. [88] report a reduction in water content in the brains of animals following ischemia with resveratrol and EGCG respectively, but it is not clear if the edema that was measured was of the vasogenic or cytotoxic type. Likewise AP1, a polyphenolic compound, also reduced brain edema in rats following MCAO [110] but the type of edema assessed is not clear. A reduction in BBB damage and water content in the brain following cerebral ischemia in rats was reported with curcumin [204]. Curcumin also decreased brain edema in rats following MCAO [205] but as with some other studies the type of edema examined is not clear. In a rat thromboembolic stroke model, curcumin reduced brain edema [206] most likely of vasogenic type. In addition, curcumin was reported to significantly lower oxidized proteins and interleukin-1β, a pro-inflammatory cytokine, elevated in the brains of AD transgenic mice [207]. Il-1 receptor 1 (IL-1R1)-null mice when subjected to hypoxia-ischemia showed reduced cytotoxic and vasogenic edema when compared to wild-type mice [208]. Taken together it is conceivable that curcumin could attenuate vasogenic edema following ischemia. Further, anti-inflammatory properties of polyphenols have been reported in other stresses and this knowledge can be applied to vasogenic edema in ischemia. Polyphenols found in cinnamon also have anti-inflammatory effects *in vitro* [209,210]. A reduction in TNF-α, an inflammatory cytokine, has been reported for green tea polyphenols [211] as well as dried plum polyphenols [212] and TNF-α is one agent that increases endothelial permeability in vasogenic edema. Also, increases in intercellular adhesion molecule (ICAM-1) and myeloperoxidases in rodent lung injury are attenuated by green tea polyphenols [213]. In addition, anti-cyclooxygenase 2 effects of resveratrol [214], as well as anti-MMP9 effects of resveratrol [215] and other polyphenols have been demonstrated. The importance of inflammation in vasogenic edema, taken together with the anti-inflammatory effects of polyphenols, indicates that the polyphenols may play a protective role in reducing vasogenic brain edema in ischemia.

## 6. Conclusion

Oxidative stress and mitochondrial dysfunction are key features of cerebral ischemia that affect neuronal viability after ischemia. In addition, these factors also affect brain edema which is a major consequence of ischemia and can be fatal if not resolved. Edema can further aggravate neuronal injury by affecting cerebral perfusion. Currently, there are few remedial agents to effectively reduce neuronal death or brain edema not only in ischemia but also in other neural injuries including traumatic brain injury. The potential for the use of polyphenols in the preventing cell loss or damage and edema in cerebral ischemic injury is tremendous. However, the cellular and molecular actions of polyphenols involved in neuroprotection have to be elucidated further. Given the large proportion of the population affected by stroke and traumatic brain injury, and with few strategies to effectively attenuate brain edema and associated neural damage, it is important to determine the potential beneficial effects of dietary polyphenols in the prevention and alleviation of such damaging effects.

## Abbreviations

AIF: apoptosis inducing factor
AP1: 5,7,3′,4′,5′-pentahydroxy dihdroflavanol-3-*O*-(2″-*O*-galloyl)-beta-d-glucopyranoside
AQP4: Aquaporin 4
ATP: adenosine triphosphate
BBB: Blood-brain barrier
CPE: Cinnamon polyphenol extract
CsA: Cyclosporin A
CSF: Cerebrospinal fluid
EGCG: epigallocatechin-3-gallate
ESAM: Endothelial cell–selective adhesion molecule
GTE: green tea extract
IAP: Inhibitor of apoptosis
ICAM-1: Inter-cellular adhesion molecule 1
IL-1β: Interleukin-1β
JAM: Junctional adhesion molecule
LPS: Lipopolysaccharide
MCAO: middle cerebral artery occlusion
MMP: Matrix metalloproteinase
mPT: mitochondrial permeability transition
NCX: Na^+^/Ca^2+^ exchanger
NHE: Na^+^/H^+^ exchanger
NKCC: Na-K-Cl co-transporter
NO: Nitric oxide
OGD: Oxygen-glucose deprivation
SOD: superoxide dismutase
TBI: Traumatic brain injury
TJP: Tight junction protein
TNF-α: Tumor necrosis factor α
ZO-1: Zonula occludens-1
